# It takes two to tango: A unique case of supraventricular tachycardia with dual competing accessory pathways

**DOI:** 10.1016/j.hrcr.2025.08.010

**Published:** 2025-08-14

**Authors:** Wei Sheng Jonathan Ong, Jannah Lee Tarranza, Wang Yue, Colin Yeo, Vern Hsen Tan

**Affiliations:** 1 Department of Cardiology, National Heart Centre Singapore, Singapore; 2 Department of Cardiology, Changi General Hospital, Singapore

**Keywords:** Atrioventricular reentry tachycardia, Supraventricular tachycardia, Accessory pathways, Electroanatomic mapping, Radiofrequency ablation

**Key Teaching Points**

• Dual accessory pathways are rare and may exhibit competing conduction, presenting a significant diagnostic and therapeutic challenge to the electrophysiologist.
• Results of traditional pacing maneuvers can be misleading in the presence of dual accessory pathways and require careful examination and validation.
• Electroanatomic mapping is critical for accurately identifying and localizing the sites of dual accessory pathways and guiding effective and targeted ablation.

## Introduction

Dual competing accessory pathways are an uncommon electrophysiological finding and can present significant diagnostic and therapeutic challenges. We describe a case of dual competing accessory pathways that were successfully identified and ablated, highlighting the complexity and nuances involved in their management.

## Case report

### Case history

A 48-year-old woman with no significant medical history presented with recurrent episodes of palpitations. She described them as sudden in onset, lasting for hours, and associated with occasional lightheadedness. She reported no chest pain or syncope. Physical examination was unremarkable, and she was hemodynamically stable during the episodes.

**Figure 1:**
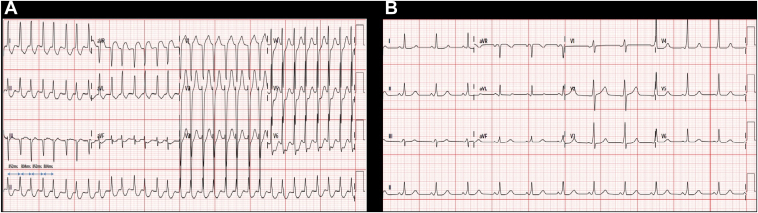
**A:** Electrocardiogram during the tachycardia. **B:** Baseline electrocardiogram.

A 12-lead electrocardiogram (ECG) recorded during an episode of palpitations (Figure 1A) revealed a narrow complex tachycardia with a short RP interval (90 ms) and alternating R-R intervals. Administration of intravenous adenosine terminated the tachycardia with a terminal P wave.

Her baseline sinus ECG (Figure 1B) demonstrated preexcitation, characterized by a short PR interval and a delta wave, and was suggestive of a left-sided accessory pathway. The transthoracic echocardiogram was normal.

In view of her recurrent symptomatic supraventricular tachycardia and suggestive ECG features, we proceeded with an electrophysiology study (EPS) for further evaluation and potential catheter ablation.

### EPS

The EPS was performed under moderate sedation. Three diagnostic catheters were introduced via the right femoral vein: an Inquiry decapolar catheter (Abbott) was positioned in the coronary sinus (CS); a Supreme CRD-2 quadripolar catheter (Abbott) was positioned at the His bundle region; and a Supreme Josephson quadripolar catheter (Abbott) was positioned at the right ventricular (RV) apex. All intracardiac signals were acquired and displayed on an electrophysiological recording system (CardioLab, GE Healthcare).

**Figure 2:**
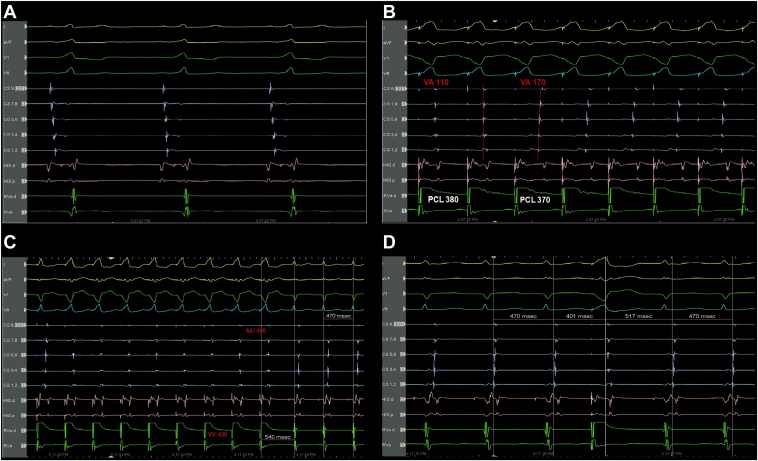
**A:** The HV interval was −15 ms. **B:** Two distinct retrograde atrial activation patterns during decremental right ventricular pacing. **C:** Ventricular overdrive pacing performed showing a V-A-V response, a post-pacing interval minus tachycardia cycle length of 70 ms, and a stimulus-to-atrial minus VA interval of 40 ms. **D:** His-refractory premature ventricular contractions advance the immediate atrial electrogram via a bystander pathway (refer to text for explanation).

Baseline intracardiac intervals were measured, and the HV interval was noted to be −15 ms (Figure 2A), indicating preexcitation through an accessory pathway.

A retrograde conduction study was performed, and it demonstrated non-decremental ventriculoatrial (VA) conduction via an accessory pathway with the earliest atrial activation at CS 1,2. The retrograde effective refractory period (without isoprenaline) of the accessory pathway was 290 ms. Interestingly, 2 distinct retrograde atrial activation patterns were observed during decremental RV pacing, both with atrial activation occurring earlier than the His atrial electrogram, suggesting the presence of 2 separate left-sided accessory pathways. One pattern showed the earliest atrial activation at CS 5,6, while the other was earliest at CS 1,2 (Figure 2B).

An antegrade conduction study was then performed. Incremental pacing from CS 9,10 demonstrated non-decremental conduction via the manifest left-sided accessory pathway. The antegrade effective refractory period (without isoprenaline) of the accessory pathway was 280 ms. The antegrade effective refractory period (without isoprenaline) of the atrioventricular (AV) node was 260 ms.

The tachycardia was reproducibly induced using single atrial extrastimuli (600/300 ms). The tachycardia cycle length was 470 ms (VA interval 100 ms), with the earliest atrial activation at CS 1,2. It demonstrated characteristics consistent with orthodromic AV reentrant tachycardia (oAVRT) using a left-sided anterolateral accessory pathway, including a V-A-V response, a post-pacing interval minus tachycardia cycle length (PPI − TCL) of 70 ms, and a stimulus-to-atrial (SA) minus VA interval (SA − VA) of 40 ms (Figure 2C). His-refractory premature ventricular contractions (PVCs) appeared to advance the next atrial electrogram (Figure 2D). However, on closer examination, there was a change in atrial activation sequence after the PVC, indicating that the premature beat likely conducted retrogradely via a separate bystander left-sided accessory pathway. As such, the His-refractory PVC maneuver did not confirm participation of the stimulated pathway in the reentrant circuit and should not be interpreted as such.

The decision was then made to proceed with electroanatomic mapping and catheter ablation.

### Mapping and ablation of the left-sided accessory pathways

Transseptal puncture was performed under intracardiac echocardiography guidance to access the left atrium. The HD Grid mapping catheter (Abbott) was then used in conjunction with the EnSite X EP System (Abbott) for high-resolution electroanatomic mapping.

**Figure 3:**
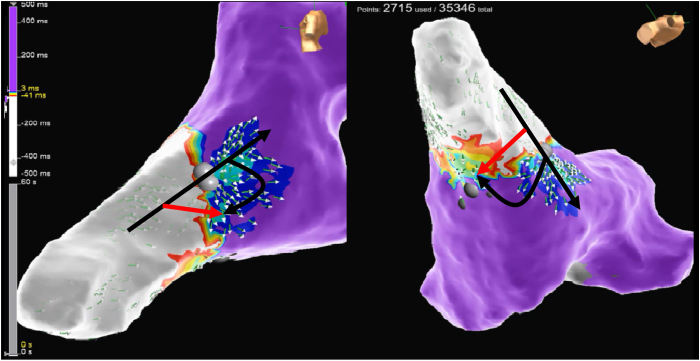
Electroanatomic map showing the orthodromic atrioventricular reentrant tachycardia using the left anterolateral accessory pathway (*black arrow*) and passive conduction via another accessory pathway located at the left posterior mitral annulus (*red arrow*).

During oAVRT, activation mapping using the open-window method identified the earliest retrograde atrial activation at the left lateral mitral annulus, corresponding to the 2 o’clock position (Figure 3). A second VA connection was also noted at the posterior mitral annulus, near the 6 o’clock position.

**Figure 4:**
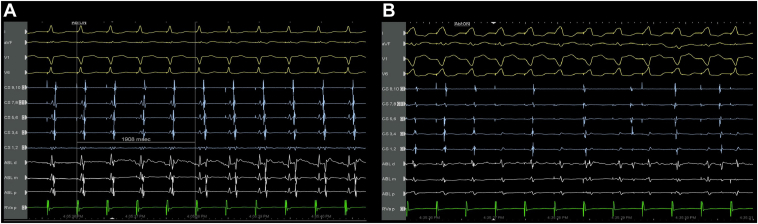
**A:** Radiofrequency ablation delivered on the left anterolateral accessory pathway (mitral annulus 2 o’clock), with termination of the tachycardia abruptly after 1.9 seconds. **B:** Radiofrequency ablation delivered on the left posterior accessory pathway (mitral annulus 6 o’clock), with successful elimination of VA conduction abruptly after 0.7 seconds.

Radiofrequency ablation was delivered at the 2 o’clock position (30 W) via the TactiCath, an irrigated contact force–sensing ablation catheter (Abbott). Ablation was performed during RV pacing with pseudofusion to maintain catheter stability and avoid abrupt catheter movement in the event of tachycardia termination. The tachycardia terminated abruptly after 1.9 seconds of energy delivery (Figure 4A). The subsequent electrogram tracing demonstrated continued RV pacing with retrograde concentric atrial activation (CS 9,10 to CS 1,2).

After successful ablation of the first accessory pathway, a second tachycardia was induced. This tachycardia also had a tachycardia cycle length of 470 ms (VA interval 90 ms) but demonstrated the earliest atrial activation at CS 3,4. This was also identified as oAVRT, this time involving a concealed accessory pathway located posteriorly on the left side. Entrainment from the RV showed a PPI − TCL of 100 ms, confirming the diagnosis. The retrograde effective refractory period (without isoprenaline) of the accessory pathway was 380 ms.

Activation mapping revealed the site of VA conduction at the 6 o’clock position along the mitral annulus. A radiofrequency ablation lesion (30 W) was delivered at this site during RV pacing, resulting in the successful elimination of VA conduction within 0.7 seconds (Figure 4B).

### Post-ablation findings and outcome

Thirty minutes after the last ablation procedure, extensive testing was performed to confirm the success of the procedure. VA dissociation was observed during RV pacing, even with isoprenaline infusion, indicating acute successful elimination of retrograde accessory pathway conduction. During testing, an AV nodal slow pathway was noted, as evidenced by the presence of an AH jump. However, as no AV nodal reentrant tachycardia could be induced, no ablation was performed. At the end of the procedure, the HV interval had normalized to 50 ms.

**Figure 5:**
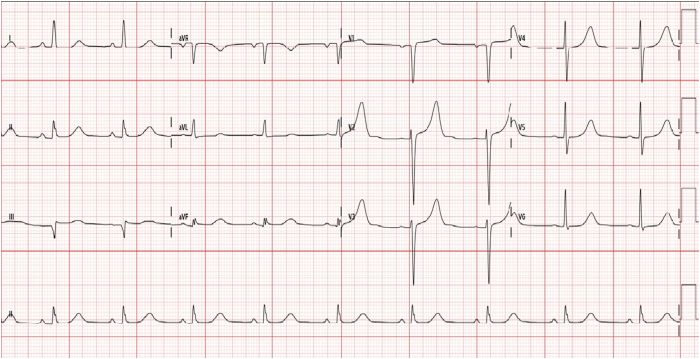
Post-ablation electrocardiogram.

The patient remained stable and asymptomatic post-procedure (Figure 5) and was discharged well, with no recurrence of tachycardia noted on subsequent follow-ups.

## Discussion

Based on the existing literature, dual accessory pathways are rare, occurring in 3%–8% of patients with AV reentrant tachycardia (AVRT),^1^, ^2^, ^3^ whereas coexistence of dual AV nodal physiology has been reported in 8%–40% of patients with accessory pathways.^4^ Our patient had a manifest bidirectional left anterolateral accessory pathway and a concealed left posterior accessory pathway, each capable of independently sustaining oAVRT. Our case underscores the importance of having a comprehensive, multifaceted approach to diagnosis and ablation in patients with multiple accessory pathways.

A particularly notable finding in our case was the presence of alternating cycle lengths, as seen on the presenting ECG. This phenomenon, however, was not reproducible during the sustained tachycardia during the EPS. The ECG also demonstrated prominent QRS alternans, an uncommon but striking feature, which strongly suggests involvement of a retrograde accessory pathway in the tachycardia circuit.^5^

We propose that the most likely mechanism underlying this phenomenon is alternating antegrade conduction between the fast and slow AV nodal pathways, supported by the presence of dual AV nodal physiology demonstrated during the EPS. This has been described in the literature as a cause of cycle length alternation during AVRT, attributed to varying AH intervals.^4^ Notably, such alternation may occur even in the absence of inducible AV nodal reentrant tachycardia, as was the case in our patient.

A less likely alternative mechanism is alternating retrograde conduction via the 2 accessory pathways. Both the manifest anterolateral and concealed posterior pathways demonstrated retrograde conduction and were independently capable of sustaining oAVRT. During decremental RV pacing, we observed alternating retrograde atrial activation sequences, suggesting the potential for dynamic switching between the 2 pathways. However, this was observed only during ventricular pacing at varying cycle lengths, and not during ventricular pacing at a constant rate. Therefore, while conceptually plausible, this mechanism is considered a less likely explanation for the ECG findings.

Nonetheless, the dynamic interaction between the 2 distinct AVRT circuits has important implications for the interpretation of standard pacing maneuvers.

A fundamental diagnostic maneuver in EPS is the measurement of the *PPI − TCL* during ventricular entrainment.^6^^,^^7^ In oAVRT, a PPI − TCL of ≤115 ms suggests that the pacing site is within or close to the reentrant circuit. However, entrainment from the RV apex may result in a delay in conduction to left lateral ventricular pathways and could lead to erroneous conclusions, though not in our case. Therefore, one should remain mindful of this potential pitfall when applying these parameters in similar contexts. In dual competing accessory pathways, ventricular entrainment can also engage a bystander accessory pathway not involved in the ongoing circuit. Hence, we interpreted the PPI − TCL together with other diagnostic maneuvers to increase the accuracy of our diagnosis.

One such supportive maneuver is the *SA − VA*, defined as the difference between the SA interval during entrainment and the VA interval during the tachycardia.^6^^,^^7^ A short SA − VA value (<85 ms) strongly suggests oAVRT. In our patient, an SA − VA of 40 ms reinforced the diagnosis. However, as was the case in PPI − TCL measurement, it is important to note that in the presence of *dual accessory pathways*, ventricular pacing may inadvertently engage a different accessory pathway than the one involved in the tachycardia, potentially leading to *misleading SA − VA values*. Thus, SA − VA values should not be interpreted in isolation.

*His-refractory PVCs* are another valuable diagnostic tool.^8^ When delivered during the tachycardia, a His-refractory PVC that *advances the atrium* without altering the His signal suggests that the *ventricle is part of the circuit* and that retrograde conduction is occurring via an accessory pathway. However, advancement of the atrium alone does not confirm that the pathway is participating in the reentrant circuit, as this can occur via a bystander accessory pathway. True participation is confirmed only if the PVC either terminates the tachycardia without retrograde atrial activation or causes a delay in the subsequent atrial electrogram, indicating interaction with the circuit itself. In our case, the PVC advanced the atrium via a bystander pathway, falsely suggesting circuit participation.

It is thus evident that in the presence of multiple accessory pathways, no single maneuver is definitive for diagnosis, and each needs to be carefully examined for validity. Accurate interpretation requires the integration of multiple parameters, including atrial activation sequences, PPI − TCL, SA − VA, and the response to His-refractory PVCs.^9^ Each provides valuable insights, but only when considered together can they lead to the most probable diagnosis.

Electroanatomic mapping remains the gold standard for resolving ambiguous findings in complex scenarios involving multiple accessory pathways.^10^, ^11^, ^12^, ^13^ We concurrently used activation mapping using the open-window method, successfully localizing 2 discrete VA connections—anterolateral and posterior—along the mitral annulus. This allowed for precise ablation at both sites, with successful termination of both tachycardias and elimination of preexcitation.

After ablation, the patient’s ECG showed no evidence of preexcitation, but her corrected QT interval was noted to be 489 ms. We believe this was due to transient repolarization abnormalities after the abrupt cessation of her chronic, incessant tachyarrhythmia. This phenomenon, known as tachycardia-induced repolarization remodeling,^14^ is typically self-limited, with normalization of repolarization within days to weeks as the myocardium recovers. Her corrected QT interval normalized to 420 ms on follow-up. To our knowledge, there is no established association between the prolonged QT interval and the presence of multiple accessory pathways.^15^

## Conclusion

This case highlights the diagnostic and therapeutic complexities posed by *dual competing accessory pathways*. Traditional pacing maneuvers, such as PPI − TCL, SA − VA, and His-refractory PVCs, provide valuable insights but can be misleading in the setting of multiple accessory pathways. Careful interpretation and examination of validity are crucial. Electroanatomic mapping proved instrumental in clarifying the arrhythmic substrate and guided successful ablation of both manifest and concealed accessory pathways. Our case demonstrates the importance of a comprehensive integrative approach to accurately diagnosing and managing complex AVRT mechanisms.

## Disclosures

The authors have no conflicts of interest to disclose.
